# Supplementary material for: Molecular basis of sidekick-mediated cell-cell adhesion and specificity
Source: eLife. 2016 Sep 19;5:e19058. doi: 10.7554/eLife.19058 (PMC5045292; doi:10.7554/eLife.19058)
Supplement: Figure 2—source data 1. — DOI: http://dx.doi.org/10.7554/eLife.19058.005 [file elife-19058-fig2-data1.docx]

|  | **Sdk1_Ig1–4_ crystal form 1** | **Sdk1_Ig1–4_ crystal form 2** | **Sdk1_Ig1–5_** | **Sdk2_Ig1–4_ crystal form 1** | **Sdk2_Ig1–4_ crystal form 2** | **Sdk2_Ig1–2_/ Sdk1_Ig3–4_** | **Sdk2_Ig1–4_ H18R/N22S** |
| --- | --- | --- | --- | --- | --- | --- | --- |
| **Data collection** |  |  |  |  |  |  |  |
| Date | 7/12/2011 | 6/19/2010 | 4/5/2013 | 9/23/2013 | 10/8/2014 | 6/14/2014 | 7/19/2013 |
| Beamline | NSLS X4A | NSLS X4C | NSLS X4C | NSLS X4C | APS 24-ID-C | APS 24-ID-E | APS 24-ID-E |
| Wavelength (Å) | 1.743 | 1.005 | 0.979 | 0.979 | 0.979 | 0.979 | 0.979 |
| Space group | *C*2 | *P*3_2_21 | *P*2_1_2_1_2_1_ | *P*2_1_ | *P*2_1_2_1_2_1_ | *P*22_1_2_1_ | *I*2 |
| *Cell dimensions* |  |  |  |  |  |  |  |
| a, b, c (Å) | 163.07, 49.26, 60.73 | 158.19, 158.19, 53.53 | 70.14, 152.87, 158.97 | 55.11, 130.01, 78.39 | 82.64, 88.36, 106.55 | 78.35, 85.73, 146.35 | 110.89, 113.43, 185.91 |
| α, β, γ (°) | 90, 110.50, 90 | 90, 90, 120 | 90, 90, 90 | 90, 100.67, 90 | 90, 90, 90 | 90, 90, 90 | 90, 103.69, 90 |
| Resolution (Å) | 50.00-2.20  (2.24-2.20) | 50.00–3.20 (3.29–3.20) | 37.16–3.50 (3.69–3.50) | 39.00–2.70 (2.85–2.70) | 45.00–3.20 (3.37–3.20) | 57.83–2.70 (2.85–2.70) | 64.84–2.70 (2.85–2.70) |
| R_merge_ | 0.100 (0.487) | 0.147 (0.520) | 0.132 (0.304) | 0.099 (0.487) | 0.122 (0.460) | 0.144 (0.502) | 0.101 (0.546) |
| I/σI | 12.2 (2.4) | 8.2 (1.5) | 9.9 (3.9) | 12.2 (2.8) | 7.1 (2.4) | 7.5 (3.1) | 10.1 (2.5) |
| Completeness (%) | 99.4 (100.0) | 90.7 (72.9) | 89.9 (79.5) | 98.7 (98.5) | 95.5 (96.5) | 99.9 (100.0) | 100.0 (100.0) |
| Redundancy | 6.8 (6.6) | 4.5 (3.6) | 3.3 (2.7) | 5.1 (5.1) | 3.9 (3.8) | 3.8 (3.9) | 3.6 (3.6) |
| **Refinement** |  |  |  |  |  |  |  |
| Resolution (Å) | 38.19−2.20 | 34.74–3.20 | 36.99−3.50 | 39.00−2.70 | 44.78–3.20 | 45.37–2.70 | 39.52–2.70 |
| Number of reflections | 22884 | 11639 | 19853 | 29407 | 12640 | 27743 | 61442 |
| R_work_ / R_free_ (%) | 19.1 / 22.8 | 20.7 / 24.4 | 20.2 / 25.1 | 19.3 / 23.0 | 20.6 / 24.8 | 19.3 / 23.3 | 20.6 / 24.3 |
| Molecules in ASU | 1 | 1 | 2 | 2 | 2 | 2 | 4 |
| *Number of residues* |  |  |  |  |  |  |  |
| Protein | 379 | 379 | 950 | 739 | 740 | 753 | 1478 |
| Carbohydrate | 4 | 6 | 13 | 7 | 8 | 4 | 12 |
| Small molecule | 0 | 0 | 2 | 1 | 0 | 3 | 0 |
| Ion | 7 | 6 | 3 | 12 | 0 | 2 | 14 |
| Water | 148 | 6 | 6 | 38 | 7 | 37 | 62 |
| *R.m.s. deviations* |  |  |  |  |  |  |  |
| Bond lengths (Å) | 0.007 | 0.002 | 0.003 | 0.003 | 0.003 | 0.004 | 0.004 |
| Bond angles (°) | 0.880 | 0.489 | 0.613 | 0.705 | 0.713 | 0.715 | 1.064 |
| *Ramachandran* |  |  |  |  |  |  |  |
| Favored (%) | 98.18 | 96.30 | 96.72 | 99.18 | 98.90 | 97.99 | 98.71 |
| Allowed (%) | 1.82 | 3.70 | 3.07 | 0.82 | 1.10 | 2.01 | 1.29 |
| Outliers (%) | 0.00 | 0.00 | 0.21 | 0.00 | 0.00 | 0.00 | 0.00 |
| Wilson B | 37.56 | 70.26 | 62.67 | 49.32 | 79.02 | 32.99 | 44.76 |
| Overall B | 45.03 | 75.68 | 64.80 | 62.20 | 77.57 | 33.95 | 60.67 |
| PDB ID | 5K6U | 5K6V | 5K6W | 5K6X | 5K6Y | 5K6Z | 5K70 |

### Figure 2—source data 1. X-ray crystallography data collection and refinement statistics

Values in parentheses are for the outer shell. ASU = asymmetric unit; R.m.s. = Root mean square.
