# Supplementary material for: Molecular basis of sidekick-mediated cell-cell adhesion and specificity
Source: eLife. 2016 Sep 19;5:e19058. doi: 10.7554/eLife.19058 (PMC5045292; doi:10.7554/eLife.19058)
Supplement: Figure 4—source data 1. — DOI: http://dx.doi.org/10.7554/eLife.19058.010 [file elife-19058-fig4-data1.docx]

| **Sidekick 1 sequences** | | **Sidekick 2 sequences** | |
| --- | --- | --- | --- |
| **Accession** | **Species** | **Accession** | **Species** |
| XP_686686.3 | Danio rerio | XP_006029532.1 | Alligator sinensis |
| XP_016122898.1 | Sinocyclocheilus grahami | XP_004860637.1 | Heterocephalus glaber |
| XP_006637301.2 | Lepisosteus oculatus | XP_008165868.1 | Chrysemys picta bellii |
| XP_012671777.1 | Clupea harengus | XP_015090733.1 | Vicugna pacos |
| XP_014801724.1 | Calidris pugnax | XP_004749092.1 | Mustela putorius furo |
| XP_010073520.1 | Pterocles gutturalis | XP_011585064.1 | Aquila chrysaetos canadensis |
| XP_010390686.1 | Corvus cornix cornix | XP_009465259.1 | Nipponia nippon |
| XP_011577492.1 | Aquila chrysaetos canadensis | XP_007482760.1 | Monodelphis domestica |
| XP_009318308.1 | Pygoscelis adeliae | XP_015735584.1 | Coturnix japonica |
| XP_010568603.1 | Haliaeetus leucocephalus | XP_006970501.1 | Peromyscus maniculatus bairdii |
| XP_008496763.1 | Calypte anna | XP_013154663.1 | Falco peregrinus |
| KFU86669.1 | Chaetura pelagica | XP_005350737.1 | Microtus ochrogaster |
| XP_014466304.1 | Alligator mississippiensis | XP_003768600.1 | Sarcophilus harrisii |
| KFQ99860.1 | Nipponia nippon | NP_766388.2 | Mus musculus |
| KFO91422.1 | Buceros rhinoceros silvestris | XP_010719784.1 | Meleagris gallopavo |
| XP_014436664.1 | Pelodiscus sinensis | XP_011287640.1 | Felis catus |
| XP_007065402.1 | Chelonia mydas | XP_006247755.1 | Rattus norvegicus |
| XP_015732346.1 | Coturnix japonica | XP_013364113.1 | Chinchilla lanigera |
| EOB00921.1 | Anas platyrhynchos | XP_005597275.1 | Equus caballus |
| KFP83217.1 | Apaloderma vittatum | XP_014644694.1 | Ceratotherium simum simum |
| XP_009090333.1 | Serinus canaria | XP_011965263.1 | Ovis aries musimon |
| XP_002190771.2 | Taeniopygia guttata | XP_014695185.1 | Equus asinus |
| XP_014122801.1 | Zonotrichia albicollis | AAN15076.1 | Gallus gallus |
| XP_005435700.1 | Falco cherrug | XP_012517683.1 | Propithecus coquereli |
| XP_005420504.2 | Geospiza fortis | XP_010340778.1 | Saimiri boliviensis boliviensis |
| XP_005145354.2 | Melopsittacus undulatus | XP_006886456.1 | Elephantulus edwardii |
| XP_009562886.1 | Cuculus canorus | XP_015666575.1 | Protobothrops mucrosquamatus |
| XP_005054485.1 | Ficedula albicollis | XP_004275482.1 | Orcinus orca |
| XP_015498549.1 | Parus major | XP_007185669.1 | Balaenoptera acutorostrata scammoni |
| KFM12390.1 | Aptenodytes forsteri | XP_511658.3 | Pan troglodytes |
| XP_014725986.1 | Sturnus vulgaris | XP_003813403.1 | Pan paniscus |
| XP_007498480.1 | Monodelphis domestica | XP_008010104.1 | Chlorocebus sabaeus |
| XP_014108198.1 | Pseudopodoces humilis | XP_007454451.1 | Lipotes vexillifer |
| NP_989436.2 | Gallus gallus | XP_011717998.1 | Macaca nemestrina |
| XP_005229039.1 | Falco peregrinus | XP_013002520.1 | Cavia porcellus |
| KFR12275.1 | Opisthocomus hoazin | XP_004041123.1 | Gorilla gorilla gorilla |
| KFZ56915.1 | Caprimulgus carolinensis | XP_014975718.1 | Macaca mulatta |
| KFQ05823.1 | Leptosomus discolor | XP_010719785.1 | Meleagris gallopavo |
| XP_004386013.1 | Trichechus manatus latirostris | NP_001138424.1 | Homo sapiens |
| XP_006143656.1 | Tupaia chinensis | XP_005584885.1 | Macaca fascicularis |
| KFP21781.1 | Egretta garzetta | XP_015135354.1 | Gallus gallus |
| XP_005621203.1 | Canis lupus familiaris | XP_015999084.1 | Rousettus aegyptiacus |
| KFV61434.1 | Picoides pubescens | XP_014133117.1 | Falco cherrug |
| XP_004394197.2 | Odobenus rosmarus divergens | XP_012418340.1 | Odobenus rosmarus divergens |
| XP_005397914.2 | Chinchilla lanigera | XP_004709379.1 | Echinops telfairi |
| XP_008017012.1 | Chlorocebus sabaeus | XP_005530779.2 | Pseudopodoces humilis |
| XP_003469953.1 | Cavia porcellus | XP_008010105.1 | Chlorocebus sabaeus |
| XP_518946.3 | Pan troglodytes | XP_016158177.1 | Ficedula albicollis |
| NP_689957.3 | Homo sapiens | XP_015501623.1 | Parus major |
| XP_004045095.1 | Gorilla gorilla gorilla | XP_016072154.1 | Miniopterus natalensis |
| XP_007901677.1 | Callorhinchus milii | XP_008154494.1 | Eptesicus fuscus |
| XP_011742520.1 | Macaca nemestrina | XP_014129483.1 | Zonotrichia albicollis |
| XP_012494361.1 | Propithecus coquereli | ELW68360.1 | Tupaia chinensis |
| KFV75189.1 | Struthio camelus australis | XP_012376244.1 | Dasypus novemcinctus |
| XP_007941083.1 | Orycteropus afer afer | XP_003495730.1 | Cricetulus griseus |
| XP_008017011.1 | Chlorocebus sabaeus | KFO30522.1 | Fukomys damarensis |
| XP_011812394.1 | Colobus angolensis palliatus | XP_007641438.1 | Cricetulus griseus |
| XP_011832325.1 | Mandrillus leucophaeus | XP_008054805.1 | Tarsius syrichta |
| EHH17116.1 | Macaca mulatta | XP_006754687.1 | Myotis davidii |
| EHH51984.1 | Macaca fascicularis | XP_015445099.1 | Pteropus alecto |
| XP_003795492.1 | Otolemur garnettii | XP_012588441.1 | Condylura cristata |
| NP_808547.3 | Mus musculus |  |  |
| XP_004671382.2 | Jaculus jaculus |  |  |
| XP_008696690.1 | Ursus maritimus |  |  |
| XP_006859935.1 | Chrysochloris asiatica |  |  |
| XP_006889806.1 | Elephantulus edwardii |  |  |
| XP_014962337.1 | Ovis aries musimon |  |  |
| XP_007174719.1 | Balaenoptera acutorostrata scammoni |  |  |

### Figure 4­—source data 1. Protein amino acid sequences used to generate Sdk sequence logos
