# Supplementary material for: Molecular basis of sidekick-mediated cell-cell adhesion and specificity
Source: eLife. 2016 Sep 19;5:e19058. doi: 10.7554/eLife.19058 (PMC5045292; doi:10.7554/eLife.19058)
Supplement: Supplementary file 1. — (A) Sidekick interdomain angles. (B) Structural comparison of IgSF protein horseshoe structures. DOI: http://dx.doi.org/10.7554/eLife.19058.019 [file elife-19058-supp1.docx]

### Supplementary file 1. Sdk and other horseshoe IgSF protein characteristics

| **Protein** | **Chain** | **Ig14:Ig23 (°)** | **Ig1:Ig2 (°)** | **Ig2:Ig3 (°)** | **Ig3:Ig4 (°)** | **Ig1:Ig4 (°)** | **Ig4:Ig5 (°)** |
| --- | --- | --- | --- | --- | --- | --- | --- |
| Sdk1_Ig1–4_ crystal form 1 | A * | 30.0 | 40.6 | 23.2 | 27.8 | 27.8 |  |
| Sdk1_Ig1–4_ crystal form 2 | A * | 37.1 | 43.5 | 23.1 | 33.1 | 35.6 |  |
| Sdk1_Ig1–5_ | A * | 31.7 | 42.8 | 23.4 | 28.3 | 30.4 | 54.3 |
|  | B | 35.5 | 42.7 | 23.2 | 33.9 | 31.5 | 54.0 |
| Sdk2_Ig1–4_ crystal form 1 | A | 31.2 | 40.5 | 14.3 | 30.8 | 29.5 |  |
|  | B * | 30.9 | 42.3 | 18.2 | 30.8 | 27.4 |  |
| Sdk2_Ig1–4_ crystal form 2 | A * | 26.2 | 42.5 | 18.8 | 26.7 | 27.4 |  |
|  | B | 26.0 | 41.5 | 17.4 | 35.7 | 27.9 |  |
| Sdk2_Ig1–2_/ Sdk1_Ig3–4_ | A | 31.7 | 37.4 | 19.3 | 20.4 | 27.4 |  |
|  | B * | 22.1 | 41.5 | 22.7 | 26.7 | 26.9 |  |
| Sdk2_Ig1–4_ H18R/N22S | A | 24.7 | 45.3 | 23.3 | 19.5 | 32.7 |  |
|  | B | 19.3 | 44.2 | 24.9 | 17.8 | 29.2 |  |
|  | C * | 24.6 | 41.9 | 19.1 | 18.5 | 30.7 |  |
|  | D | 21.4 | 43.6 | 15.9 | 18.7 | 31.1 |  |
| *Average* |  | *28.0 ± 5.4* | *42.2 ± 1.9* | *20.5 ± 3.3* | *26.3 ± 6.3* | *29.7 ± 2.5* | *54.2 ± 0.2* |

### Supplementary file 1A. Sidekick interdomain angles

Ig14:Ig23 is the angle between the Ig1:Ig4 and Ig2:Ig3 planes in the horseshoe. Ig1:Ig2 is the angle between the Ig1 and Ig2 axes, defined by mass weighting. *Indicates chain shown in Figure 2A or Figure 4–figure supplement 3A.

| **Protein** | **Ig14:Ig23**  **(°)** | **Ig1:Ig2**  **(°)** | **Ig2:Ig3**  **(°)** | **Ig3:Ig4**  **(°)** | **Ig1:Ig4**  **(°)** | **RMSD to Sdk1** |
| --- | --- | --- | --- | --- | --- | --- |
| Sdk1 | 34 | 42 | 23 | 31 | 31 | N/A |
| Sdk2 | 29 | 42 | 17 | 31 | 28 | 1.0 Å |
| Dscam^PDB: 3DMK^ | 10 | 69 | 43 | 30 | 30 | 5.8 Å |
| CNTN2^PDB: 2OM5^ | 8 | 43 | 21 | 13 | 29 | 3.4 Å |
| CNTN4^PDB: 3JXA^ | 15 | 51 | 30 | 14 | 33 | 2.8 Å |
| DCC^PDB: 3LAF^ | 36 | 82 | 40 | 6 | 44 | 5.5 Å |
| Neurofascin^PDB: 3P3Y^ | 14 | 50 | 23 | 7 | 35 | 4.8 Å |
| Hemolin^PDB: 1BIH^ | 20 | 55 | 27 | 7 | 31 | 6.2 Å |
| *Average* | *21 ± 11* | *54 ± 14* | *28 ± 9* | *17 ± 11* | *33 ± 5* |  |

### Supplementary file 1B. Structural comparison of IgSF protein horseshoe structures

Average horseshoe protein interdomain angles over all chains in all crystal structures are given. Ig14:Ig23 is the average angle between the Ig14 and Ig23 planes. The right hand column gives the root mean square deviation over aligned Cα atoms (RMSD) between the Ig1–4 structures of the various IgSF proteins and Sdk1. The Ig1:Ig4 angle is most rigid among horseshoes, highlighting the importance of Ig1:Ig4 interface in horseshoe conformations.
